# A Quarter of a Century of Medicine

**Published:** 1891-08-08

**Authors:** 


### The Hospital

A Weekly Journal of

Science, Medicine, Nursing, and Philanthropy.

*For Hospitals, Asylums, Infirmaries, Dispensaries, Medical Practitioners, Students, Nurses, and the Charitable Public.*

Vol. X.—No. 254.] [Aug. 8, 1891.

### A Quarter of a Century of Medicine.

The deliberate reader of Dr. Lauder Brunton's address in medicine to the members of the British Medical Association last week, has forced upon his intelligence the conviction that medicine during the last quarter of a century has been making an epoch in the world's history. Medical science is almost the latest-born of intellectual forces; but it is mighty, and it promises to be mightier. Show us your results, say the doubters, and we will believe. The beginnings of results are evident to those who are on the field of research; the promise of great and striking marvels is clear and distinct. But outsiders can as yet only see the confusion and smoke of battle; they will reap the fruits of conquest in due time.

Medicine has accepted the leadership of Bacon. She does not look for the knowledge of man, of physical man, in the clouds; she inspects man himself. As Dr. Lauder Brunton says, there are many things so complex that they cannot be comprehended in the mass, by the ordinary intelligence; they have to be taken in detail. The synthesis is there before our eyes; but we have not made it, and we are not masters of it. We must make our analysis first; and it must be complete. When we have mastered every detail we can recompose; and we can then say, we know.

It is frequently said that cancer is on the increase, and much unnecessary care and anxiety are thereby inflicted upon nervous men and women. Science, that is true science, enables us to contradict the distressing statement. Dr. Lauder Brunton points out that the present generation of medical men know diseases when they see them, and so give them their right names. A former generation, guessing at them, very frequently assigned death to wrong causes. When we see, therefore, that more people seem to die of certain diseases now, and fewer of certain other diseases, than was formerly the case, we are not entitled to say generally that the incidence of disease has changed, but that the true factors which produce death are more generally recognised than they formerly were. Cancer is a case in point. "I am quite certain," says Dr. Brunton, "that many cases which were formerly classed as chronic diarrhœa, dysentery, jaundice, or dropsy, were really due to malignant disease (that is cancer) of the abdomen, while others probably depended upon unrecognised disease of the kidney."

This undoubted fact of more competent diagnosis serves to show how medicine has gradually entered upon a path of profounder knowledge and wider power. When men had found out what diseases were, their next inevitable step was to ask why they were? The modern physiological laboratory, with all that it includes, is the concrete answer to that question. What is consumption? What is Cancer? What is Typhoid Fever? Whence do they come? Why do they seize upon the human organism and destroy it? We want answers to these questions. Where shall we look for those answers? It is plain that we must look, at least in the first instance, to the organisms themselves in which the diseases appear. We must go further and search out the very parts of the human body which are specially attacked. But we cannot do this in the living subject; we cannot bring our microscopes and other exploring instruments to bear upon a breathing tuberculous lung or a living cancerous kidney. We have to wait until life is gone. But the dead-house can never reveal anything but dead secrets; it can show us nothing but the completed work of destruction. What we want is knowledge of disease in the living. We must see diseased processes not only when they have brought about destruction, but in their earlier stages. Nay, if we are to perform our highest functions as physicians we must get our eyes upon them at the very outset. We must go a step further back even than this, and learn, whenever it is possible, to recognise the cause of the disease, before it has approached the commencement of its destructive operations.

It is clear, then, that if medicine is to be a science, if the healing art is ever to do its greatest work, we must know at least these two things: the nature and progress of disease, and the action and power of remedies in the living body. But, as it has been pointed out, we cannot employ our most effective searching instruments upon living men and women. Failing living men and women, there are other creatures, similar to them in most respects, which suffer from similar diseases, or which can have similar diseases induced in them by artificial means. Dr. Brunton affirms that the sources of our knowledge are the normal and abnormal physiological processes of living animals, as those processes have been scientifically observed by all the methods which are at the command of the vivisector.

The knowledge gained in the physiological laboratory is of two kinds; it is first a knowledge of diseased processes, their causes, beginnings, continuous history, and terminations: and it is, secondly, a knowledge of the effect of various methods used with the view of preventing diseases altogether, of checking them in their course, or of modifying them in their consequences. Now, these two kinds of knowledge are the most fundamental of all the conditions of rational medical treatment. No reasonable person will question this proposition. Dr. Brunton takes it for granted that there is no other possible method of gaining these two kinds of knowledge but that of vivisection. He takes his stand upon the indispensable necessity for the two kinds of knowledge, and also upon what by many will be considered a still higher ground, the approbation of the greatest of all the teachers of mankind. "Ye are of more value than many sparrows," said the Founder of Christianity. "How much, then, is a man better than a sheep?" said the same Divine Teacher. From these principles Dr. Brunton contends that man may use sparrows, sheep, and other animals for any purpose whatever which is distinctly serviceable to the whole human race. Moreover, he argues that the knowledge obtained by vivisection is not of service to man alone, but to the lower animals. They have their full and proper share of all the benefits which spring from the knowledge gained in the physiological laboratory. The few animals which suffer in the cause of science do not suffer for man alone, but for all other sentient creatures of every class and degree.

The method of minute and patient investigation of actual facts, and the rigid scrutiny and critical judgment of definite results is the method above all others which modern medicine employs, and the method above all others which leads to actual and assured progress. It is the final and ultimate method of science, and it is the method which must be applied to every department of human life and activity if science and civilization are to advance. Religion and politics must, and inevitably will, be put into this crucible. In both politics and religion men will insist upon asking, Whence comes a particular institution? Why is it here? What are its fruits? How can it be conserved if it be good? How can it be modified if it be partly good and partly evil? Or, How can it be destroyed if it be bad? In this campaign of science medicine leads the way; and at the present moment at any rate, the physiological laboratory leads medicine. It is not to be wondered at that timid and sensitive spirits resist the onward march of this revolutionary force; still less is there cause for surprise that those souls which are possessed with the enthusiasm of knowledge and truth should contend for the right to march forward even to the death. Be it for good, or be it for evil, medicine, the medicine of the laboratory, is in the van of science; and whether honour or defeat is to be the fate of the scientific army, it will fall, in the fullest measure, upon the pioneerforce.

The outside world must have a little patience with medical discoverers. In metaphysics, and even in theology, many people think the last word has been said: there is nothing new to discover. The case is not so with medicine. The new methods of modern days are to the old medicine what the new Christianity of the Roman Empire was to the old pagan unbeliefs and superstitions. Let the new wine do its work. When it is put into old wine-skins, we must not wonder if they burst. But if there be new wine there are new wine-skins awaiting it: new minds, new courage, new resolution. Medicine, it is probable, will never abolish death. But if it some day reaches the point of carrying every healthy child, with reasonable security, from the cradle to a full old age, it will have deserved more honour at the hands of civilised man than he is sometimes disposed to give it.

